# Supplementary material for: Primary care providers’ and nurses’ knowledge, attitudes, and skills regarding latent TB infection testing and treatment: A qualitative study from Rhode Island
Source: PLoS One. 2022 Apr 15;17(4):e0267029. doi: 10.1371/journal.pone.0267029 (PMC9012388; doi:10.1371/journal.pone.0267029)
Supplement: S1 File — (DOCX) [file pone.0267029.s002.docx]

**Key Informant Interview Guide**

*Baseline Questions*

We are conducting these interviews to understand the thoughts, comfort, and knowledge of PCPs regarding latent tuberculosis infection (known as LTBI). There are no right or wrong answers to these questions, as we are seeking to understand what Rhode Island PCPs are thinking about LTBI in order to develop useful educational programming through a new telementoring system in the state.

1) Can you please tell me a little bit about your current role in primary care?

(probing questions below)

1. Are you a physician, NP, nurse, other?
2. Do you have a patient panel?
3. Please describe your patient population.
4. What percentage of your patient panel are non-US born (this includes refugees, immigrants, undocumented patients)?
5. Do you conduct annual wellness visits/physical exams as part of your practice?

2) For a 35-year old male, which conditions would you say you most commonly screen for during an annual wellness visit?

3) Do you generally screen patients for TB infection?

1. (if yes) In a typical month, how many patients do you screen for LTBI?
2. (if not) Why don’t you screen patients?

4) How do you decide which patients need TB infection testing or a referral for TB infection testing.

- 1. What patient characteristics (or factors) would lead you to test for TB infection?
  2. Does anything get in the way of doing (referring) latent TB infection testing in patients who you think need it?
     1. (If they are overwhelmed by tests) How would you get LTBI testing back on your to do list?
  3. (If not incorporating into practice) What would help you do TB infection testing for these patients?

5) Tell me about which tests you have heard about for TB infection.

- 1. Which do you prefer? Why?
  2. Does anything make it difficult to do the tuberculin skin test?
     1. Follow up here with patients returning, timing, etc (probes)
  3. Does anything make it difficult to do the interferon gamma release assay test?
     1. Follow up here about timing, labs

6) Tell me about what you do in your current practice if someone screens positive for TB infection.

- 1. (If they just send to RISE…) Do you find that you do a symptom screen or order an xray while the patients are waiting for their RISE appointment?
  2. (if they just send to RISE…) Have you ever considered incorporating these steps into your practice?

7) Have you ever evaluated an individual for TB infection?

OR

(If has evaluated individuals for TB infection) How comfortable are you with the evaluation process which includes symptom screening and chest xray to rule out TB disease.

- 1. If very comfortable, how did you get so comfortable?
  2. If not comfortable, why is that?
  3. Are there certain individuals that you feel more comfortable evaluating? For example, certain ages or certain disease states.

8) Have you ever considered treating TB infection in your practice?

- 1. (**If NO**) Why is that?
     1. Would anything help you incorporate treatment into your practice?
  2. (**If YES**) How have you gotten to the spot where you are comfortable treating TB infection?
  3. We have heard from others that primary care providers do not often treat TB infection themselves. Why do you think that is?
     1. What are the challenges that prevent treatment?
     2. Where do these patients get treated?
     3. How does the referral process work?
  4. (**If has referred**) What communication do you have with a TB specialist regarding TB infection management for a patient? Please describe that experience.

9) (**If treating**) Which patient groups are you less comfortable treating for TB infection.

- 1. What about children? Why?
  2. What about pregnant patients? Why?
  3. What about people living with HIV? Why?
  4. Any other individuals?

10) Tell me about what treatment options you have you heard about for TB infection.

- 1. Which ones do you feel most comfortable prescribing? Why?
  2. What have you heard about the most recent recommendations for TB infection treatment?

**11) (If treating**) Tell me about how you conduct follow up visits after initiating TB infection treatment.

- 1. Are there any barriers to conducting follow up visits?

12) Do you find that you do any reporting to the department of Public Health with positive TB infection results?

13) (ONLY IF TREATING) Do you conduct any reporting to the department of public health when a patient completes TB infection treatment?

14) (If has reported TB infection) How do you feel about the reporting system to the department of public health regarding TB patients?

1. Ask about feelings about reporting system in general?

15) How have you learned about TB infection testing and treatment?

- 1. What topics or skills would you like to have more training on related to latent tuberculosis screening and treatment?

16) What else would you like to tell me about LTBI screening and treatment in practice that would help us develop an educational program that is helpful to Rhode Island’s PCPs?

17) By the way, The USPSTF has listed LTBI screening as a grade B recommendation. Have you heard about that?
