# Supplementary material for: Primary care providers’ and nurses’ knowledge, attitudes, and skills regarding latent TB infection testing and treatment: A qualitative study from Rhode Island
Source: PLoS One. 2022 Apr 15;17(4):e0267029. doi: 10.1371/journal.pone.0267029 (PMC9012388; doi:10.1371/journal.pone.0267029)
Supplement: Attachment — Submitted filename: Review and proposals_B.Neziri_27621.pdf [file pone.0267029.s003.pdf]

Review and proposals

Manuscript Number: PONE-D-21-05814
Article Type: Research Article
Full Title: Primary care providers' and nurses' knowledge, attitudes, and skills regarding latent TB infection testing and treatment: A qualitative study from Rhode Island

I read carefully the paper with title and manuscript list above.

Beside that authors rise a thematic that is very import for healthcare institutions and hospitals, I consider that the proposal paper lacks in many aspects it orders to meet the scientific requirements for publication, especially in a journal as PLOS One is.

According to my opinion and the main concern is that the number of 21 primary care providers (PCP) including in this quality study is not sufficient, in a population of Rhode Island of more than 1 Mio, and a number of physicians of more than 2500. Also, I consider that the authors, should prepare one questionnaire that is uniform and all participants in the study to answer on it and not to be based just on the interview. I consider that on this approach the authors could get much more reliable data's for their scientific study and based on these to have much more clear picture for knowledge, attitudes and skills of PCP regarding LTBI in Rhode Island.

In Introduction part there is many sentences that has not a reference; Line 62-65; Line 71-80; Line 84-86; ect...

The Results part has not been clarifying fully in all study aspect and moreover is not present appropriately. There is just one table and some interviews.

The Reference part is not uniform. I'm not sure for the style the authors selected and if this style has been applying to all references. See references Nb. 6,7,8,9,10,11,...

Also, the English written style in some sentences was not clear and understand, so my proposal is to be check by any professional scientific experience researchers for improve.

Based on all point mention above, I considered that this paper should be revise completely and after to be submitted once more.

Thank you and best regards.

Prof.Dr. Burim Neziri

University of Prishtina

Medical Faculty
