# Supplementary material for: Primary care providers’ and nurses’ knowledge, attitudes, and skills regarding latent TB infection testing and treatment: A qualitative study from Rhode Island
Source: PLoS One. 2022 Apr 15;17(4):e0267029. doi: 10.1371/journal.pone.0267029 (PMC9012388; doi:10.1371/journal.pone.0267029)
Supplement: Attachment — Submitted filename: PlosOne.RevisionResponses.FINAL.docx [file pone.0267029.s004.docx]

Journal Requirements:

When submitting your revision, we need you to address these additional requirements.

1. Please ensure that your manuscript meets PLOS ONE's style requirements, including those for file naming. The PLOS ONE style templates can be found at
   <https://journals.plos.org/plosone/s/file?id=wjVg/PLOSOne_formatting_sample_main_body.pdf> and 
   <https://journals.plos.org/plosone/s/file?id=ba62/PLOSOne_formatting_sample_title_authors_affiliations.pdf>

**Thank you for this comment. We have carefully reviewed the style requirements and edited our manuscript accordingly.**

2. Please provide additional details regarding participant consent. In the ethics statement in the Methods and online submission information, please ensure that you have specified what type you obtained (for instance, written or verbal, and if verbal, how it was documented and witnessed). If your study included minors, state whether you obtained consent from parents or guardians. If the need for consent was waived by the ethics committee, please include this information.
Once you have amended this/these statement(s) in the Methods section of the manuscript, please add the same text to the “Ethics Statement” field of the submission form (via “Edit Submission”).

For additional information about PLOS ONE ethical requirements for human subjects research, please refer to <http://journals.plos.org/plosone/s/submission-guidelines#loc-human-subjects-research>.

**We have added a detailed description of our participant consent process in the methods and online submission form.**

2. Please consider including more information on the number of interviewers, their training and characteristics; and please provide the interview guide used.

**Thank you for this suggestion. We have added details about the two interviewers and have added the interview guide as supplemental material.**

4. Thank you for stating in your Funding Statement:
 
"DS is partially supported by Institutional Development Award Number U54GM115677 from the National Institute of General Medical Sciences of the National Institutes of Health, which funds Advance Clinical and Translational Research (Advance-CTR <https://www.brown.edu/initiatives/translational-research/home>). SK is supported by NIH/NIAID under R25AI140490 – Emerging Infectious Disease Scholars (EIDS <https://www.brown.edu/academics/medical/plme/current-students/enrichment-activities/research-opportunities/emerging-infectious-diseases-scholars->) Program at Brown University.
The content is solely the responsibility of the authors and does not necessarily represent the official views of the National Institutes of Health, Advance-CTR, or EIDS."

Please provide an amended statement that declares *all* the funding or sources of support (whether external or internal to your organization) received during this study, as detailed online in our guide for authors at <http://journals.plos.org/plosone/s/submit-now>.  Please also include the statement “There was no additional external funding received for this study.” in your updated Funding Statement. 
Please include your amended Funding Statement within your cover letter. We will change the online submission form on your behalf.

**Thank you. We have followed the instructions and added the requested information regarding funding.**

5. Please include captions for your Supporting Information files at the end of your manuscript, and update any in-text citations to match accordingly. Please see our Supporting Information guidelines for more information: <http://journals.plos.org/plosone/s/supporting-information>

**Thank you. We have done so.**


[Note: HTML markup is below. Please do not edit.]

Reviewers' comments:

Reviewer's Responses to Questions

5. Review Comments to the Author

Reviewer #1: Thank you for your valuable work. The following will be effective in improving your work.

1. Explaining the necessity of working with a qualitative method has not been done, while the questions can be answered with a quantitative method.

**Thank you. In the introduction, we explained in our final paragraph that the aim of this study was to explore the knowledge, attitudes, and skills among RI PCPs and nurses regarding the latent TB infection care cascade. Since this exploration has not been done before, and PCPs’ knowledge, attitudes, and skills were not previously known, we did not feel a quantitative approach would be the best option since we did not yet know the hypotheses we wanted to study further. For exploration into this topic, asking study participants to tell us in their own words through in-depth qualitative interviewing provided information that can form the basis for future quantitative study and contribute to development of highly relevant survey questions and response categories. Therefore, insights from this qualitative study can form the basis for future quantitative work to be undertaken by our group or other researchers.**

2. There is a need to edit and justify the lines.

**Our manuscript is left justified with longer quotes indented, as is typical for qualitative manuscripts. We apologize that we do not understand this suggestion. We would be happy to reformat upon receiving more detailed instruction.**

3. What was the criterion for completing sampling and saturation in this study?

**Thank you. In the Participant Recruitment section, we have added additional information about our sampling criteria. In the Data Collection section, we added information about how we reached data saturation.**

4. Which method was used to analyze the data? (With reference)

**We provide additional details in the Data Analysis section regarding our two analysis approaches - Immersion/Crystallization for analytical reading, and Template Organizing Style for coding. References 14 and 16 refer to these analysis techniques.**

5. How is the data confidentiality criterion met?

**Thank you. Transcripts were cleaned and any identifiable data were removed. We added this clarification to data analysis.**

6. Explain the inclusion and exclusion criteria.

**Please see the participant recruitment section for an expanded explanation of this.**

7. Explain the questions used in the research (At least the basic questions)

**Thank you. We have included our entire interview guide as supplemental material (S2 File).**

8. Category tagging has happened with quantitative and pre-arranged visibility.

**For this qualitative study, we used data analysis techniques that are widely used in qualitative research. First, we used immersion/crystallization to closely read the transcripts; then we used the Template Organizing Style approach to use the notes taken during our close ready to develop a codebook and code the qualitative data. The final step was to use the code reports generated by the NVivo qualitative coding software to conduct further immersion/crystallization on the coded data segments to further categorize the findings into themes. References number 14 and 16 are citations for these qualitative data analysis approaches.**

Reviewer #2: This a wonderful study and is aimed at promoting ending of TB disease through not missing any LTBI untreated. It is very vital that this approach to ending TB is widely known and utilised by all countries main with high TB stats.

In the background it was noted that there was no mention of LTBI treatment regimens as to be comprehensive in the clear description of the problem at hand.

**We have added the LTBI treatment regimens most commonly used in the US to the introduction.**

There is also a need to describe the distinct difference between the PCPs and Nurses as these causes confusion as to who is who. This is given the notion that nurses are primary health care provided and this is at least what is known universally.

**Thank you for this important point. We have added an explanation of the common tasks performed by PCPs vs nurses in the US. Nurses are not primary health care providers in the US as they are in other countries.**

The article was well formatted

Reviewer #3: Manuscript Number: PONE-D-21-05814
Article Type: Research Article
Full Title: Primary care providers’ and nurses’ knowledge, attitudes, and skills regarding latent TB
infection testing and treatment: A qualitative study from Rhode Island

I read carefully the paper with title and manuscript list above.

Beside that authors rise a thematic that is very import for healthcare institutions and hospitals, I consider that the proposal paper lacks in many aspects it orders to meet the scientific requirements for publication, especially in a journal as PLOS One is.
According to my opinion and the main concern is that the number of 21 primary care providers (PCP) including in this quality study is not sufficient, in a population of Rhode Island of more than 1 Mio, and a number of physicians of more than 2500.

**Thank you for this comment. We can understand that the reviewer is likely coming from a quantitative perspective, and agree that for a quantitative study, our sample size of 21 PCPs and 3 nurses would not be sufficient. However, our study is not quantitative; it is a qualitative study. Qualitative samples are always far smaller than quantitative samples. There is no formula to calculate the appropriate sample a priori – rather, the concept of data saturation is used to determine when we had reached an adequate number of interviews. When interviewers noted the same answers again and again and no new themes were emerging, we ceased data collection. For recruitment, we used purposive sampling and ensured that we had PCPs interviewed from large Federally Qualified Health Centers in the state that serve at-risk populations.**

Also, I consider that the authors, should prepare one questionnaire that is uniform and all participants in the study to answer on it and not to be based just on the interview. I consider that on this approach the authors could get much more reliable data’s for their scientific study and based on these to have much more clear picture for knowledge, attitudes and skills of PCP regarding LTBI in Rhode Island.

**Thank you for this comment. We chose to conduct a qualitative study rather than a quantitative study for a number of important reasons. Given that the exploration of knowledge, attitudes, and skills of PCPs regarding LTBI had never previously been studied, we chose a qualitative methodology over a quantitative questionnaire since structured, close-ended questions had the potential to incorporate an information bias as well as limit data received. For example, one of our open-ended questions in our interview guide asked how PCPs decide which patients need TB infection testing or a referral for TB infection testing (question 4 in S2 File). In order to convert this to a close-ended question, we would have needed to have potential answer choices which were unknown to us at the time and were not in the literature. We agree with the reviewer that at this point in time, using the findings from our study, one could follow up with a larger quantitative survey and derive questions for this survey from our qualitative data.**

In Introduction part there is many sentences that has not a reference; Line 62-65; Line 71-80; Line 84-86; ect…

**Thank you for this important comment. We have added three references in the introduction to support some of the sentences highlighted.**

The Results part has not been clarifying fully in all study aspect and moreover is not present appropriately. There is just one table and some interviews.

**Unlike for quantitative papers where the bulk of data analysis is often presented in tables, this is not the case for qualitative papers. It is typical in journal articles based on qualitative data to include illustrative quotes from the interviews embedded within the narrative or placed in tables. It is more informative to include the quotes within the text so that clarity is enhanced about exactly what analysis points the quotes are meant to illustrate. In our results, therefore, we included a table of participant characteristics only, as further tables are not needed since the analysis and quotes were included in the narrative text. In addition, we included a figure that summarizes the overall themes.**

The Reference part is not uniform. I’m not sure for the style the authors selected and if this style has been applying to all references. See references Nb. 6,7,8,9,10,11,…

**Thank you. We have revised the references to ensure uniformity.**

Also, the English written style in some sentences was not clear and understand, so my proposal is to be check by any professional scientific experience researchers for improve.

**We are unclear about which parts of the paper the reviewer found to be unclear or ungrammatical. All authors on this study are native English speakers, with our senior author having published close to 100 peer-reviewed manuscripts in English. Please provide us with specific details about which sections of the paper need improvement in the writing style.**

Based on all point mention above, I considered that this paper should be revise completely and after to be submitted once more.
Thank you and best regards.
