# Supplementary material for: Primary care providers’ and nurses’ knowledge, attitudes, and skills regarding latent TB infection testing and treatment: A qualitative study from Rhode Island
Source: PLoS One. 2022 Apr 15;17(4):e0267029. doi: 10.1371/journal.pone.0267029 (PMC9012388; doi:10.1371/journal.pone.0267029)
Supplement: Attachment — Submitted filename: PLOSONE.R2.ReviewerResponses.FINAL.docx [file pone.0267029.s005.docx]

Response to Reviewers

6. Review Comments to the Author

Reviewer #1: Thank you for your valuable work. The following can be effective in improving your work
The necessity of the work and the reason for using the qualitative method should be highlighted in the introduction.

**Thank you for this comment. We have added justification for the work as well as using qualitative methodology in the introduction.**

- Explain the method of analysis and the type of qualitative method.

**This study used key informant interviews with PCPs and nurses in Rhode Island who work with at risk populations. A justification for this type of method has been added to the method section.**

**The methods of analysis are described in the data analysis section and involved two steps – immersion/crystallization and template organizing style. We recognized that we accidentally included the immersion/crystallization reference in the wrong place and have updated this.**

- Explain how to achieve saturation

**The last line of the data collection paragraph describes how we reached data saturation. Reference 13 supports our approach.**

- Explain how to ensure study rigor.

**Our stepwise data analysis approach, the use of Nvivo, and our check-ins with all co-authors ensured study rigor. We have added a sentence reflecting this at the end of data analysis.**

- Labeling the categories with a qualitative look

**Our process of using immersion/crystallization for familiarity with content and to develop the code categories for the next phase of template style analysis using NVivo coding is outlined in the analysis section. With this method, we coded for content and then went back into the coded data, again using immersion/crystallization techniques, to identify patterns and themes in the coded data to come to final interpretation.**

- At the beginning of the method, the clinical trial code is stated, but this study is descriptive and qualitative

**This manuscript describes the results of the first aim of a three-phase exploratory sequential translational study. Aim 2 is a trial. As described in detail when the clinical trial # is searched (**[**https://clinicaltrials.gov/ct2/show/NCT04188041)**](https://clinicaltrials.gov/ct2/show/NCT04188041))**, the results of the qualitative study described in this manuscript informed the design of the educational intervention studied in Aim 2. We did not provide additional details about this since the methods are outlined carefully on the trial website.**
